# Facile Synthesis of Amine-Functionalized Eu^3+^-Doped La(OH)_3_ Nanophosphors for Bioimaging

**DOI:** 10.1007/s11671-010-9768-x

**Published:** 2010-09-02

**Authors:** Conroy Sun, Colin Carpenter, Guillem Pratx, Lei Xing

**Affiliations:** 1 Department of Radiation Oncology, Stanford University School of Medicine, 875 Blake Wilbur Drive, Room G206, Stanford, CA 94305-5847, USA

**Keywords:** Nanophosphor, Nanoparticle synthesis, Lanthanum hydroxide, Europium, Photoluminescence, Bioimaging

## Abstract

Here, we report a straightforward synthesis process to produce colloidal Eu^3+^-activated nanophosphors (NPs) for use as bioimaging probes. In this procedure, poly(ethylene glycol) serves as a high-boiling point solvent allowing for nanoscale particle formation as well as a convenient medium for solvent exchange and subsequent surface modification. The La(OH)_3_:Eu^3+^ NPs produced by this process were ~3.5 nm in diameter as determined by transmission electron microscopy. The NP surface was coated with aminopropyltriethoxysilane to provide chemical functionality for attachment of biological ligands, improve chemical stability and prevent surface quenching of luminescent centers. Photoluminescence spectroscopy of the NPs displayed emission peaks at 597 and 615 nm (λ_ex_ = 280 nm). The red emission, due to ^5^D_0_ → ^7^F_1_ and ^5^D_0_ → ^7^F_2_ transitions, was linear with concentration as observed by imaging with a conventional bioimaging system. To demonstrate the feasibility of these NPs to serve as optical probes in biological applications, an in vitro experiment was performed with HeLa cells. NP emission was observed in the cells by fluorescence microscopy. In addition, the NPs displayed no cytotoxicity over the course of a 48-h MTT cell viability assay. These results suggest that La(OH)_3_:Eu^3+^ NPs possess the potential to serve as a luminescent bioimaging probe.

## Introduction

Lanthanide-doped phosphors have been investigated and employed in a broad range of technological applications such as solar energy converters, fluorescent lighting and high-performance displays [1,2]. Recently, nanophosphors (NPs) have received significant attention for use in biological sensing [3-5] and imaging applications [6,7] due to their unique physical and optical properties. As fluorescent probes, similar to semiconductor quantum dots (QDs), these luminescent NPs offer several advantages over conventional organic fluorophores, including high photochemical stability, large Stokes shift and tunable fluorescence emission [8]. In addition, these rare-earth-based nanoparticles exhibit several distinct and useful characteristics for monitoring molecular and biological interactions [8,9]. Newly developed NPs exhibit long fluorescence lifetimes, no photoblinking and lower cytotoxicity, which make them attractive alternatives to QDs.

The unique photophysical properties of trivalent lanthanide (Ln^3+^) ions, which act as luminescence activators in these NPs, result from electronic transitions within the 4f shell. Electrons in these 4f orbitals are shielded from interaction with the chemical environment by the 5s and 5p shells, which are lower in energy, but spacially located outside of the 4f orbitals [10]. This unique electronic configuration gives rise to emission spectra that are characterized by sharp peaks, whose spectral positions are independent of their matrix. However, host materials play a critical role influencing the fine structure and emission intensities by absorbing excitation light and transferring energy to the Ln^3+^ ions. In addition, this matrix can reduce non-radiative relaxation, such as the O–H vibration of water, which results in luminescence quenching [11,12]. Lanthanide chelates that bind Ln^3+^ ions and incorporate organic chromophores, which serve as antenna or sensitizers, have been developed as bioprobes for time-resolved luminescent (TRL) immunoassays and Forster resonant energy transfer (FRET) experiments [13-15]. Although versatile, these organic complexes are subject to photochemical degradation, which remains a major challenge in the development of these biological probes.

In response to a growing demand for more sensitive and robust fluorescence bioimaging agents, inorganic Ln^3+^-doped NPs composed of rare-earth oxides, oxysulfides, phosphates and fluorides are currently being actively investigated [8,9]. In these NP platforms, the host matrices tightly bind and sensitize a variety of Ln^3+^ ions allowing for a wide range of emission colors through selection of dopants. One of the most widely used phosphor activators is trivalent europium (Eu^3+^) due to its well-characterized emission spectrum resulting from ^5^D_J_(*J* = 0,1,2) → ^7^F_J_(*J* = 0,1,2,3) transitions. The use of stable Eu_2_O_3_ is limited by high cost and poor luminescence due to strong concentration quenching [16]. In order to improve Eu^3+^ emission, various rare-earth host NPs have been synthesized by modification of conventional phosphor fabrication processes, such as solid-state and pyrolysis reactions [17,18]. Although efficient in producing highly luminescent NPs, these synthesis methods typically require specialized equipment and can suffer from particle aggregation limiting their use under physiological conditions. Recent efforts to produce NPs for biological applications have focused on colloidal synthesis methods capable of producing dispersed water-soluble NPs. In addition, surface modification of NPs to improve biocompatibility and allow for conjugation of biological ligands continues to be actively investigated [19-21].

In this study, we present a facile synthesis process to produce ultrafine Eu^3+^-doped NPs for use as luminescent bioimaging probes. Here, lanthanum hydroxide is utilized as a rare-earth matrix material due to its lack of inherent luminescence [10] and straightforward conversion to oxides or oxysulfides through dehydration or sulfuration [22]. This wet chemical strategy employs a low-molecular weight poly(ethylene glycol) (PEG) as a high-boiling point solvent allowing for monodispersed NP formation and in situ polymer coating. The physically adsorbed amphililic PEG serves as a temporary barrier to particle aggregation and allows for convenient solvent exchange for subsequent surface modification in either aqueous or organic solutions. Here, we demonstrate surface coating with aminopropyltriethoxysilane (APTES) to improve chemical stability and provide amine functional groups for conjugation of biomolecules. As-synthesized and APTES-coated NPs were characterized to determine their physiochemical and photoluminescence properties. Functionalized NPs were also evaluated as luminescent probes in a conventional biological imaging system and in vitro fluorescence microscopy. In addition, an initial assessment of the biocompatibility of these NPs was performed using a MTT cell viability assay.

## Experimental

### Materials

Lanthanum nitrate hexahydrate (La(NO_3_)_3_·6H_2_O), poly(ethylene glycol) (PEG, MW 570-630), titianum (IV) isopropoxide Ti(OPr)_4_ (3-aminopropyl)trimethoxysilane (APTES), dimethyl sulfoxide (DMSO) and MTT (3-(4,5-Dimethylthiazol-2-yl)-2,5-diphenyltetrazolium bromide) were purchased from Sigma–Aldrich (St. Louis, MO). Europium oxide (Eu_2_O_3_), nitric acid (HNO_3_) and toluene were purchased from Acros Organics (Morris Plains, NJ). Sodium hydroxide (NaOH) and 200 proof ethanol (EtOH) were purchased from Fisher Chemical (Fairlawn, NJ). All reagents were used without further purification. Deionized (D.I.) water was obtained from an ultrapure water purification system (Barnstead Nanopure, Thermo Scientific, Dubuque, IA). Dulbecco's modified Eagle's medium (DMEM), fetal bovine serum (FBS), phosphate-buffered saline (PBS), penicillin/streptomycin, TrypLE and Trypan blue were purchased from Gibco (Invitrogen, Carlsbad, CA).

### Nanophosphor Synthesis and Surface Modification

Eu^3+^-doped NPs were obtained through a high-temperature precipitation in low-molecular weight PEG. Initially, 39 mg Eu_2_O_3_ was dissolved in 0.2 ml 35% HNO_3_ under magnetic stirring. Then, 1.9 g La(NO_3_)_3_·6H_2_O was dissolved in 0.8 mL of D.I. water and combined with the dissolved Eu_2_O_3_ and 40 mL of PEG. A volume of 1.0 mL of 6 M NaOH was then added dropwise under vigorous stirring, and the mixture was heated to 140°C for 1 h. The reaction vessel was then sealed and heated for 3 h at 140°C. The resulting colloidal solution was cooled to room temperature and diluted 1:10 in EtOH. To isolate the NPs, 50-mL aliquots of the NPs mixture were centrifuged at ~2,500 RPM for 5 min. The supernatant was decanted to remove excess PEG and reaction byproducts. NPs were washed twice by redispersing them in 50 mL of EtOH followed again by centrifugation and decanting the supernatant. The particles were then resuspended in 50 mL of anhydrous toluene. Surface modification was performed with the addition of 0.5 mL APTES and 50 μL Ti(OPr)_4_, as a catalyst, for 12 h at 40°C in an ultrasonic bath (Branson Ultrasonic Corp., Danbury, CA). The coated NPs were centrifuged, and excess APTES and Ti(OPr)_4_ were decanted. The resulting NPs were purified by washing three times with ethanol and D.I. water, respectively.

### Physiochemical Characterization of Nanophosphors

Transmission electron microscopy (TEM) samples were prepared by dipping 400-mesh copper grids (Veco, Ted Pella, Redding, CA) in a diluted suspension of NPs in EtOH. The grids were then dried and imaged on a JEOL TEM1230 (Tokyo, Japan) operating at 80 kV. Digital TEM images were acquired with a Gatan 967 (Pleasanton, CA) slow scan, cooled CCD camera. Particle size distribution was calculated using NIH ImageJ with the particle size analyzer (PSA) macro. Powder X-ray diffraction (XRD) patterns were acquired from dried samples with a PANalytical X'Pert (Almelo, Netherlands) diffractometer using Cu-Kα radiation (λ = 1.541Å) at 40 kV and 20 mA. X-ray photoelectron spectroscopy (XPS) experiments were carried out using a Surface Science Instrument S-probe spectrophotometer (Newburyport, MA) with a monochromatized Al X-ray source and 5 eV flood gun for charge neutralization. X-ray spot size for the acquisition was approximately 800 μm. Pressure in the analytical chamber during spectral acquisition was less than 5 × 10^-9^ Torr.

### Photoluminescence Characterization of Nanophosphors

Photoluminescence characterization was performed on a FluoroMax-4 spectrofluorometer (Horiba Scientific, Edison, NJ). Images of NP emission intensity as a function of concentration were obtained on a Xenogen Illumina system (Caliper Life Sciences, Hopkinton, MA). For imaging, optical phantoms were prepared over a range of concentration in 1% agarose. Fluorescence emission was normalized to photons per second per centimeter squared per steradian (p/s/cm^2^/sr).

### In Vitro Imaging Assay

Human cervical adenocarcinoma HeLa cells were cultured in DMEM supplemented with 10% FBS and 1% penicillin/streptomycin at 37°C in a humidified atmosphere with 5% CO_2_. Cells were grown on 75-cm^2^ cell-culture flasks with 10 mL of media that was changed every second day. To assess the optical detection of the NPs in a biological sample, cells were incubated with the NPs at a concentration of 100 μg NP/ml for 4 h at 37°C. Cells were then washed twice with PBS and incubated with TrypLE solution (substitute for porcine trypsin) for 5 min at 37°C to detach them from the flask. Cell density was determined through staining with trypan blue and counting cells using a hemocytometer with 0.9-mm^3^ counting chamber. To evaluate NP fluorescence from bulk cell sample, 10^6^ cells were fixed in methanol at -20°C for 15 min and centrifuged to isolate a pellet for imaging. For fluorescence microscopy, cells preincubated with NPs, as described earlier, were seeded on 24-mm glass cover slips and incubated for 18 h to allow them to attach. Cells were then washed with PBS three times and fixed in methanol at -20°C for 15 min. The cover slips were then mounted on slides using Prolong Gold antifade solution with DAPI (4',6-diamidino-2-phenylindole) (Invitrogen, Carlsbad, CA) for cell nuclei staining and fluorescence preservation. The slides were examined by fluorescence microscopy using a Leica fluorescence microscope (Wetzlar, Germany) equipped with excitation/emission filter cubes for DAPI and FITC (fluorescein isothiocyanate). Here, an FITC 515-nm-long-pass emission filter was used to acquire all NP emission in the green and red spectral region. By convention, signal collected by the DAPI and FITC filter cubes were colored blue and green, respectively.

### Cytotoxicity (MTT) Assay

Human breast adenocarcinoma MCF7 cells were cultured in DMEM supplemented with 10% FBS and 1% penicillin/streptomycin at 37°C in a humidified atmosphere with 5% CO_2_. Cells were then cleaved with TrypLE and counted using a hemocytometer, as described earlier. The cells were then seeded on 96-well plates (5,000 cells/well) in DMEM w/o phenol red. After 12-h incubation, cells were incubated with APTES-coated NPs at concentrations of 0, 0.1, 1.0, 10 and 100 μg/mL in 200 μL DMEM w/o phenol red. In addition, control (cells only) and blank (no cells) samples were also present on the well plates. The cells were then incubated at 37°C for 12, 24 and 48 h. Four hours before the end of each time point, 20 μL of 12 mM MTT was added to each well. Upon completion of the incubation, all but 25 μL of the medium was removed, and 50 μL of DMSO was added to each well to dissolve the formazan crystals. The samples were then mixed thoroughly with pipetting, and the absorbance read at 540 nm on a microplate reader (Tecan, Mannedorf, Switzerland).

## Results and Discussion

### Nanophosphor Synthesis and Surface Modification

**Figure 1:**
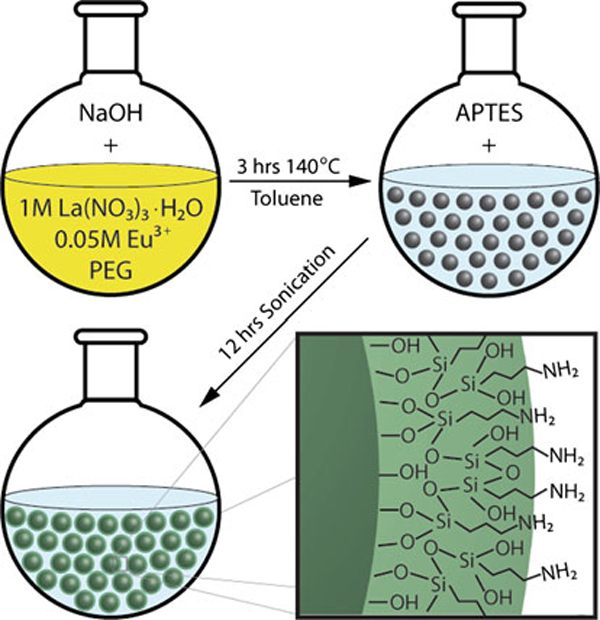
**Schematic diagram of nanophosphor synthesis in poly(ethylene glycol) (PEG) and surface modification with aminopropyltriethoxysilane (APTES)**.

**Figure 2:**
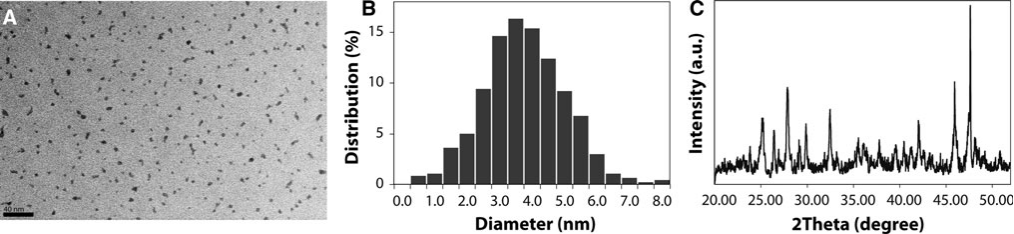
**a Nanophosphor (NP) size and morphology was evaluated by transmission electron microscopy (TEM)**. **b** Particle size distribution of NPs as determined by TEM. **c** XRD pattern of the NPs.

The overall NP synthesis and functionalization process is illustrated in Figure 1. This straightforward procedure can be carried out with general chemistry laboratory equipment and utilizes relatively low-cost reagents. In this study, the size and morphology of the NPs produced by this process were analyzed by TEM. As shown in Figure 2a, NPs are roughly spherical in shape with a mean diameter of ~3.5 nm. Measurement of particle size with the NIH ImageJ image software package displayed good uniformity and narrow size distribution (Figure 2b) of the NPs, which are significant advantages of the synthesis procedure. The size of these NPs is on the order of biomacromolecules, such as cell-surface receptors or antigens, making them ideal for molecular imaging applications. In this size range, the NPs can also be taken up by cells via endocytotic vesicles, which are typically 40–60 nm in diameter [23]. Furthermore, the small size of these NPs opens the potential for in vivo applications by overcoming the size restriction of biological barriers [24,25] and allowing for clearance to minimize long-term toxicity [26,27].

The structure and composition of the NPs were analyzed by X-ray characterization techniques. Powder XRD was performed on the APTES-coated NPs (Figure 2c). Peaks consistent with crystalline La(OH)_3_ were observed (JCPDS 36-1481); however, additional peaks were also detected and attributed to the presence of La_2_O_3_ and possibly the Eu^3+^ dopant. Furthermore, the relatively low intensity of the peaks suggests a significant amorphous phase is present and likely due to extensive reconstruction at the surface of these nanoparticles. In comparison with previous reports [28,29], the use of PEG as a surfactant in this hydrothermal synthesis of La(OH)_3_ NPs significantly reduces the overall size of the nanostructure with a trade-off of well-defined crystal structure. Previously, polyol synthesis methods employing diethylene glycol to produce Y_2_O_3_:Eu^3+^ and Gd_2_O_3_:Eu^3+^ NPs have been reported [30]. Here, we employ PEG as a more biologically friendly and solvent compatible reaction medium for surface modification. Further processing by annealing the NPs synthesized in PEG and conversion to lanthanum oxide or lanthanum oxysulfide [31] are potential areas of optimization for this NP platform; however, these processes are beyond the scope of this work.

**Figure 3:**
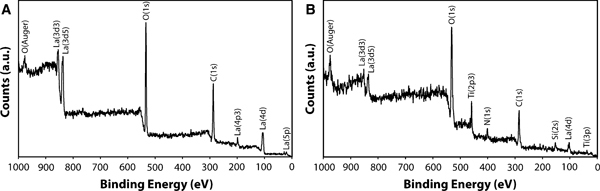
**XPS survey scan of a as-synthesized and b APTES-coated NPs**.

To confirm the NP composition and evaluate the surface modification process, both as-synthesized and APTES-coated NPs were characterized by XPS. The survey scan obtained from the as-synthesized NPs (Figure 3a) confirms the primary composition of lanthanum and oxygen with main peaks of La 3d5 and O 1s centered at ca. 835.2 and 531.1 eV, respectively. In addition, the presence of physically adsorbed PEG in these samples likely contributes to the prominent C 1 s peak. The low concentration of the Eu^3+^ dopant (<5 mol%) is below the detection limit of this technique. Confirmation of the dopant in NPs is described in the following section. The XPS survey scan of the APTES-coated NPs (Figure 3b) displays peaks characteristic of the amine-bearing silane in addition to the NP matrix. Main peaks of Si 2 s and N 1s at binding energies of 152.2 and 400.7 eV, respectively, confirm the surface functionalization of NPs. An additional peak at 458.3 eV was identified as titanium (Ti 2p3), which was also incorporated in the coating from the Ti(OPr)_4_ catalyst [32]. The presence of this siloxane bound coating improves the chemical stability and protects the Eu^3+^ luminescent centers that may be located at or near the surface of the NP from quenching by water. Furthermore, the terminal amine group serves as a convenient linking chemistry for bioconjugation [33,34], which is necessary for biological applications.

### Nanophosphor Photoluminescence

**Figure 4:**
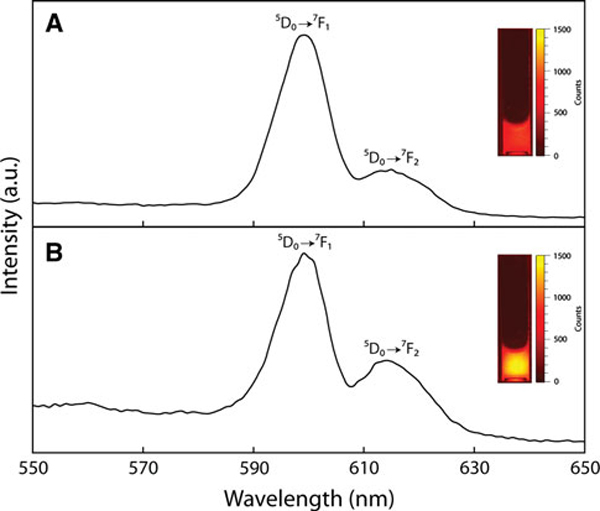
**Photoluminescence spectra of a as-synthesized NPs and b APTES-coated NPs (λ_ex_ = 280 nm)**. Images of optical phantoms containing 5 mg/mL as-synthesized NPs **a ***inset* and APTES-coated NPs, **b ***inset* (λ_em_ = 575 - 650 nm).

The optical properties of the La(OH)_3_:Eu^3+^ NPs were evaluated by fluorescence spectroscopy under UV excitation in the broad Eu–O charge transfer band [35,36]. The photoluminescence spectra of the as-synthesized NPs, shown in Figure 4a (λ_ex_ = 280 nm), display typical Eu^3+^ emission peaks at 597 and 615 nm due to the ^5^D_0_ → ^7^F_1_ (magnetic-dipole) and ^5^D_0_ → ^7^F_2_ (electronic-dipole) transitions, respectively. The emission spectra of APTES-coated NPs, shown in Figure 4b, display the same peak positions; however, the intensity of the 615-nm emission relative to the ^5^D_0_ → ^7^F_1_ is increased. The stronger ^5^D_0_ → ^7^F_2_ transition is likely due to the migration of Eu^3+^ ions to crystalline positions in the NP lattice during aging over the surface modification process. In addition, the intensity of emission in the red spectral region was enhanced by surface coating, as described previously. Analysis of the APTES-coated NP in an optical phantom (Figure 4b inset, 5 mg/mL) displayed ~44% enhancement in emission intensity when compared to bare NPs (Figure 4a inset).

**Figure 5:**
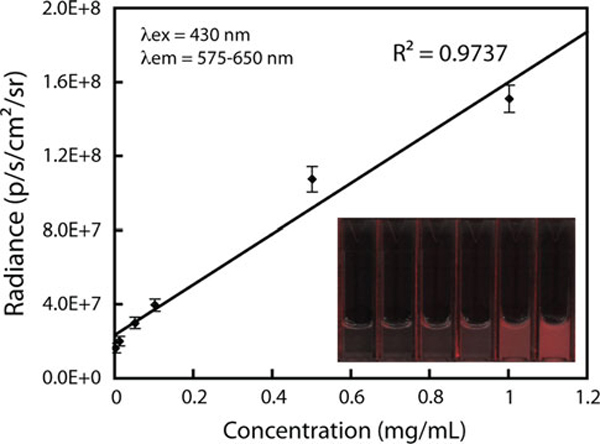
**Nanophosphor emission intensity as a function of concentration**. Image of optical phantoms containing 0, 0.01, 0.05, 0.1, 0.5 and 1.0 mg/mL NP, *inset*.

In biological imaging applications where UV radiation can damage organic structures, blue excitation has been employed with Eu^3+^ complexes [37]. To demonstrate the use of these NPs in a conventional bioimaging system, optical phantoms containing various concentrations of APTES-coated NPs (0, 0.01, 0.05, 0.1, 0.5, 1.0 mg/mL) were analyzed in an IVIS Illumina fluorescence imaging system (λex = 430 nm). The fluorescence intensity, expressed in radiance, was measured and plotted as a function of concentration (Figure 5). The NPs displayed a linear relationship of luminescence with concentration potentially enabling their use in biological sensing and imaging applications.

### In Vitro Cell Labeling

**Figure 6:**
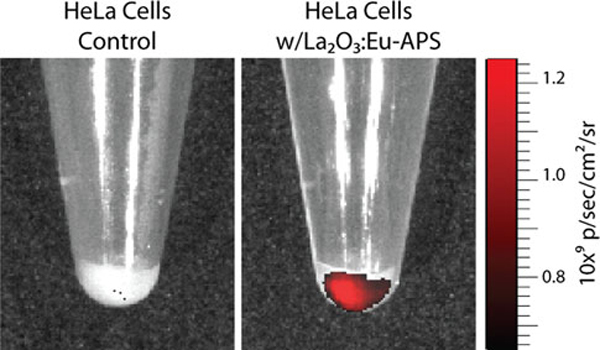
**Fluorescence images of HeLa cell pellets containing ~106 cells, control (*left*) and NP labeled (*right*), acquired with a conventional bioimaging system (Xenogen IVIS Illumina)**.

Fluorescence labeling and optical detection of molecular interactions has become an indispensable tool in biology. To demonstrate the efficacy of the amine-functionalized La(OH)_3_:Eu^3+^ NPs to serve as a luminescent probe under biological conditions, an in vitro imaging experiment was performed with human cervical cancer HeLa cells. In this assay, cells were labeled with APTES-coated NPs via charge interaction between the positive amine terminal groups and negatively charged cell membrane. In addition, non-specific uptake of NPs was likely due to the high incubation concentration (100 μg/mL) and extended incubation times (4 h). After the NP incubation, the cells were washed, cleaved, and centrifuged. The resulting cell pellet containing ~ 10^6^ cells and a control sample w/o NPs were imaged with the IVIS Illumina imaging system (Figure 6). The successful fluorescent labeling of the cells with NPs was observed by the red emission in comparison with control cells.

**Figure 7:**
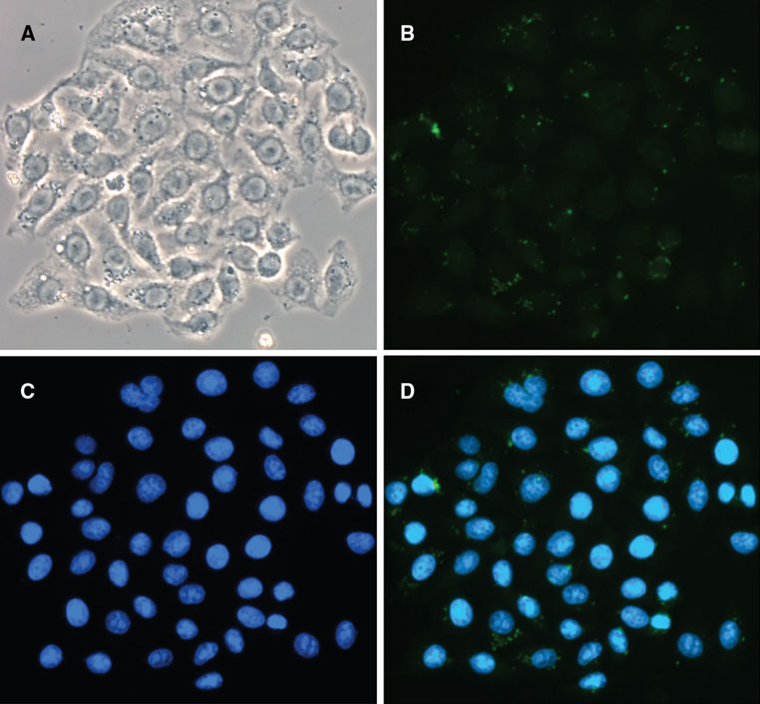
**a Phase contrast image of NP-labeled HeLa cells**. **b** Nanophosphor emission captured by FITC long-pass emission filter, which includes the red spectral region. **c** DAPI emission identifying cell nuclei. **d** overlay of **b** and **c**.

To visualize the luminescence of these NPs on the cellular level, La(OH)_3_:Eu^3+^ NP labeled HeLa cells were seeded on microscope cover slips and imaged with a fluorescence microscope (Figure 7a–d). In this in vitro fluorescence microscopy experiment, HeLa cells were stained with DAPI to identify the cell nucleus (Figure 7b, blue), while the NPs were visualized with a FITC excitation (420–490 nm)/emission (515 nm long pass) filter set (Figure 7c, green). The emission of the probes localization in the cell cytoplasm, likely within the endosomal or lysosomal compartments, demonstrates their ability to serve as optical imaging bioprobes. In addition, after incubation with the NPs, no changes in cell morphology or other visual signs of cytotoxicity were observed in the phase contrast images (Figure 7a).

### Nanophosphor Cytotoxicity

**Figure 8:**
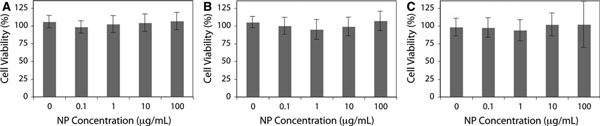
**MTT cell viability assay of MCF7 cells incubated with various concentrations of NPs over a 12 h, b 24 h and c 48 h**.

To further investigate the biocompatibility of the APTES-coated La(OH)_3_:Eu^3+^ NPs, these NPs were evaluated with human breast adenocarcinoma MCF7 cells as a model cell line. The cells were grown for intervals of 12, 24 and 48 h with a NP concentration range typically used in biological assays (0, 0.1, 1.0, 10 and 100 μg/mL). Cell proliferation was monitored with a MTT assay in which the reduction of a yellow tetrazolium salt in metabolically active cells forms purple formazan crystals [38]. The spectroscopic quantification of the formazan corresponds with cellular viability shown in Figure 8a–c. In this assay, no significant cytotoxicity was observed over the concentration entire range tested during the 48 h of the experiment. These results suggest that amine-functionalized La(OH)_3_:Eu^3+^ NPs may potentially serve as optical contrast agents for bioimaging.

## Conclusions

In this study, we have developed a facile synthesis process to produce luminescent water-soluble La(OH)_3_:Eu^3+^ NPs for biological applications. The NPs obtained from this process are ~ 3.5 nm in size and display optical emission typical for Eu^3+^-activated probes with ^5^D_0_ → ^7^F_1_ and ^5^D_0_ → ^7^F_2_ transitions. Surface functionalization with APTES was confirmed with XPS and shown to enhance luminescence intensity by ~ 44%. In addition, the amine terminal groups of the APTES-coated NPs provide reactive sites for bioconjugation. Combining the ability to attach a variety of biological ligands with selection of Ln^3+^ dopant–tuned emission, these La(OH)_3_ NPs may serve as effective multiplex imaging agents for various biological assays [39]. To demonstrate the luminescence of the NPs under biological conditions, an in vitro experiment was performed with HeLa cells. NP emission was observed in the cells by both a conventional bioimaging system and through fluorescence microscopy. In addition, the NPs displayed no apparent cytotoxicity over the course of a 48-h MTT cell viability assay making them suitable optical probes for bioimaging applications.
